# Wider Action Plan and Multidisciplinar Approach Could Be a Wining Idea in Creation of Friendly Environment

**DOI:** 10.1155/2012/473427

**Published:** 2012-02-08

**Authors:** Natasa Gojkovic-Bukvic, Nenad Bukvic

**Affiliations:** ^1^Logistics Management Consultancy, Viale Unità d'Italia No. 69, 70125 Bari, Italy; ^2^Department of Economics, LUM Jean Monnet University, S.S. 100 km18, 70010 Casamassima, Italy; ^3^Section of Cytogenetics and Molecular Biology, Department of Clinical Pathology, University Hospital, OORR Foggia, Viale Luigi Pinto No. 1, 71100 Foggia, Italy

## Abstract

Herein, we proposed planning of wide transdisciplinary actions, which bring a solution for economic activity such as transportation, strongly related to pollution output with possible repercussions on climate change and public health. To solve logistics problem by introduction of common intermodal policy, and creation of more friendly transport solution, it is possible to obtain sustainable development, climate change prevention, government policy, and regulation which are all related to human health and creation of health-supportive environment. This approach permits environmental and biological monitoring same as economic results measurement by key performance indicators. This approach implementing emerging scientific knowledge in environmental health science such as genetic epidemiology aimed at understanding how genomic variation impacts phenotypic expression and how genes interact with the environment at the population level with subsequent translation into practical information for clinicians as well as for public health policy creation.

## 1. Introduction

Economic and industrial growth in the last century provoked the massive increase of air pollutants, resulting from more intense energy consumption and exhaust emissions from vehicles, with important global environmental consequences including climate change. Undoubtedly, increase of air pollutants same as global climate change will have multiple effects on human health especially on vulnerable populations such as children, the elderly, and the poor who are at increased risk from such events [1].

The contamination of air by organic and inorganic toxic pollutants same as exposure to motor vehicle emissions represents an important concern for possible long-term health effects [2–5]. Negative associations between traffic-related pollution and respiratory health have been underlined by different epidemiological studies on air pollution [6–15]. D'Amato et al. [16] reported observations that in the regions with high levels of vehicle traffic the interplay between climate change and the most abundant components of air pollutions (airborne particulate matter, nitrogen dioxide and ozone) alters the concentration and distribution of air pollutions and as consequence interferes with the seasonal presence of allergenic pollens in the atmosphere by prolonging this period with adversely effects on lung function in asthmatics. The air pollutants with well-established respiratory effects will potentially change as climate change [17, 18] and/or could be influenced by warming temperatures which can affect chemical reactions rates [19, 20]. Traditionally, climate change has been considered as an environmental rather than a health issue.

Quantification of the effects of climate change on health is needed on all levels (global, regional, and local) through enhanced monitoring of environmental health and one of the possible ways could be biomonitoring.

Biomonitoring of the exposure to complex mixtures such as polluted ambient air, vehicle exhaust, and smoke is a particular challenge since these exposures have many constituents in common and many people were exposed to more than one of these mixtures. It is well known that human biomonitoring comprises the determination of different validated biomarkers which are generally assigned to one of three classes: biomarkers of exposure, biomarkers of effect, and biomarkers of susceptibility. Their application in epidemiological studies has been proven. Most studies used random samples of citizens with mixed activities and exposure profiles, with intention to be representative of the whole population [21]. The intensity of exposure of the study subjects could be done with passive personal samplers as well as blood and urinary biomarkers and could be also compared with ambient, for example, benzene concentrations, measured by municipal monitoring stations [22].

As an example of good practice to be followed, a report on tobacco toxicants of the Institute of Medicine (USA) could be considered. Namely, reducing risk of disease by reducing exposure to tobacco toxicants is feasible and biological markers associated with tobacco-related disease could be used to offer guidance as to whether or not Potentially Reduced Exposure Products (PREPs) are likely to be risk-reducing [23].

This observation raised a simple question: if the same could be used to perform biomonitoring of populations at places of high traffic density, with higher exposition/air pollutions and could it be measured, obviously before and after introduction of friendly environment transport project with positive consequences on human health, climate change preparedness strategy as a part of public health programs?

Climate change and adverse environment are perceived as a health threat and the solutions are necessary by different type of actions. We focus on the health impact and possibility to propose public health strategy through energy consumption reduction, implementing new intermodal transportation chain. Stronghold for this approach underlined that framework for prevention includes incorporation of climate change actions into the 10 essential functions of public health as reported by Frumkin et al. [24].

The project idea was born during attempt to find a way how to connect South Italy (South Europe, Spain, and Portugal) to South East Europe/Balkan Peninsula countries in the most suitable way, with less air pollution, more traffic safety, and reduction of road congestion. This approach should be seen as immediate implementation of the European Union Common Transport Policy and enlargement of European Union on Balkan Peninsula countries, which are still out of EU and also to establish joined traffic management, as one of the most industrialized topic areas within transport research, with consequences for public policy issues related to government regulation, human health, and/or environment. Furthermore, this could be a way to create future strategy able to “burn out” timeline gap provoked by recent historical events and to prepare Balkan Countries for the future partnership giving to the Countries from this region a possible solution to help health promotion and building up environmental systems able to avoid the contamination (discussed with great interest at REACT—conference 2011 [25]).

## 2. Climate Change and Transportation

The greenhouse gas emission (GHG) in the decade up to 2008 for the EU27 decreased by 2,4%. Energy use, waste, manufacturing, construction, and agriculture were the areas where emissions decreased but at the same time emissions from energy industries, industrial processes, and transport were growing.

**Figure 1:**
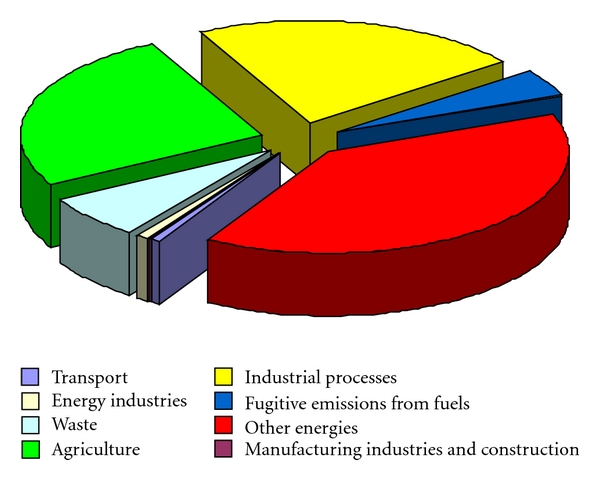
EU-27 Greenhouse Gas Emissions by sector, 2008 [26].

Figure 1 reported the most recent data published by EUROSTAT regarding the situation of GHG emissions by sector in decade up to 2008 [26].

**Figure 2:**
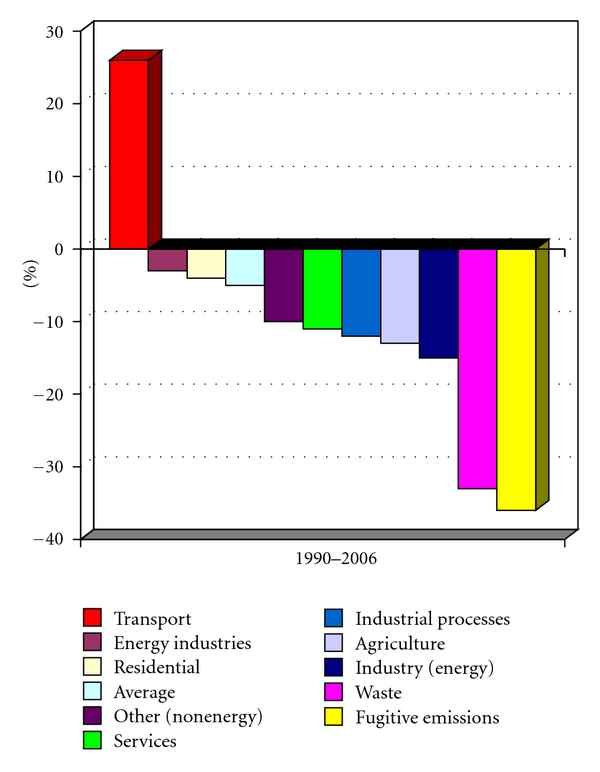
CO_2_ emissions from transportation EU-25 [27].

The increase in emission of air pollutants and climate change due to economic and industrial growth has made air quality an important problem throughout the world. These emissions give rise to climate change with increased social costs (i.e., diseases), costs that do not have to be carried by the actual polluter. The GHG emissions in EU have been reduced in most sectors over the last 15 years, beside transportation which has shown a 25% increase (Figure 2) [27].

In order to come to terms with this, many European governments have to decide to take legislative actions. The level of GHG emission is to be reduced by 40% by 2020 and by 2030 the Swedish vehicle fleet is to be fully independent of fossil fuels. The social cost will have to be internalized and to achieve this carbon taxes and emissions trading schemes will be utilized [27, 28].

The company may choose between a number of measures in order to achieve a reduction. One measure commonly suggested is a shift in transport mode, from faster, more polluting mode such as road and air transport to slower and less polluting modes such as rail or sea transport developing proper logistics chain. A particularly interesting solution is an intermodal road-rail-short sea shipping solution. In this way, the flexibility and availability of truck transport is combined with low-cost, CO_2_ efficient, rail transport for the longer part of the journey. Research has shown that, with this type of mode shift, CO_2_ emission can be reduced by 20–50% or more depending on how the energy for the train part is produced [29, 30].

Climate change is a major threat to sustainable development. On the basis of Kyoto Protocol, EU15 has a collective target of 8% of reduction below levels chosen in a base year (mostly 1990) which had decreased by 2008 by 6,9%. After that, the EU27 has set a 20% reduction target to be achieved by 2020 [31].

Transport is the second largest source of emissions in the EU and it is the sector that has exhibited continuously growing emissions [26]. A task of the EU Sustainable Development Strategy is to achieve a balanced shift towards environmentally friendly transport modes which will bring about a sustainable transport and mobility system. This shift would certainly fall GHG emissions as well towards environmental friendly transport modes.

## 3. European Union Plans for the Region

EU plans for the Region are grouped in hard and soft measures; the hard measures are related to infrastructures and soft measures are harmonization and reforms (technical standards and border crossing procedures). The soft projects that are considerably affected by “regionalization” deal with the railways that could be solved by setting up Intergovernmental Working Group on Railway and Intermodal Policy. The Working Group is to make an inventory of rail reforms and further recommend measures that ensure the regional integration and harmonization of the reforms for every country and to open access to transport infrastructure. Unfortunately, States have usually denied railways enterprises the freedom of a commercial business and this has to be changed. Different options are possible: some railways may focus entirely on their core business of operating trains. Others may choose to enter into partnership, for example, with road haulers or logistics companies and offer door to door intermodal services. Some may operate across Europe, while others may concentrate on local services. One thing in common of all railways in Region is that they must focus on what their customers want and how they can satisfy these needs. It is important to establish common traffic management which will focus on planning, monitoring, and controlling of traffic. The principal aim should be to maximize the effectiveness of the use of existing infrastructure, ensure reliable and safe operation of transport, address environmental goals, and ensure fair allocation of infrastructure space (road space, rail slots, etc.) among competing users [32].

Given the various confusions among stakeholders about the implication of the Carbon Pollution Reduction should serve to increase understanding of various effects of this initiative and possible multidisciplinary approaches regarding this theme.

## 4. Biomarkers and Adverse Human Exposure

For many years some cytogenetic alterations as biomarkers of genotoxic exposure have been used [33–38]. The fact that most established human carcinogens are genotoxic represents a relevant reason for using these assays [33]. In fact epidemiological studies strongly suggest that the high frequency of some of them is predictive of an increased risk of cancer [34, 38]. Gene polymorphisms are also able to modulate the human response to genotoxic insults [33, 39–43]. In fact any polymorphism that affects genes acting on metabolism or cellular response to DNA damage may alter individual sensitivity to genotoxic carcinogens.

From the 1950s mutagenicity testing became an important topic for geneticists who were well trained to recognize structural chromosome rearrangements (CA). Up today, different tests (SCE, MN, Comet Assay, etc.) were introduced which correlate at least with parts of metaphase chromosome aberration assays. The newest tests and approaches are faster and less laborious and do not require *a priori *knowledge about the diversity of structural chromosome aberrations. This lack of knowledge can easily lead to unnecessary misinterpretation of genomic data [44].

As exposure to environmental factors including tobacco smoke and diet may significantly affect the onset of most cancers [45], gene-environment interactions can modulate the outcome of such exposure influencing individual susceptibility to tumour initiation and development. Thus, genetic variability in metabolic activities related to some enzymatic pathways may partially explain individual susceptibility to cancer. The competition and interplay between different metabolic pathways are expected to modulate the levels and pattern of the DNA adducts and consequently modify the risk of cancer development, and in several cases the polymorphic variants have been found to confer to the encoded enzymes higher or lower capacity to activate or detoxify the genotoxic compounds [37, 46]. 

Recently microRNAs (miRNAs; small noncoding RNAs) have been suggested to be important in maintaining the lung in a disease-free state through regulation of gene expression (epigenetics mechanisms); however little is known regarding whether environmental agents can induce such changes. Observation of Jardim et al. [47] reports that alteration of miRNA expression profiles by environmental pollutants such as diesel exhaust particles (DEPs) can modify cellular processes by regulation of gene expression, which may lead to disease pathogenesis. However, mechanisms of damage by DEP exposure to human respiratory health are only partially known, as reported by Li et al. [48]. The same authors confirm upregulation of Matrix-Metalloproteinase-1 (*MMP-1*) in response to DEPs in human bronchial epithelial (HBE) cells and suggest that human = 1607GG polymorphism is a susceptibility factor for high response [48].

More research for understanding of the interplay between genetic and environmental factors is necessary, as discussed by Comuzzie [49] regarding the challenge for applied genetic epidemiology and its relation on the information obtained by completion of Human Genome Project which is to put the human genome in context. This means not only to identify genes impact but also to know how they interact with the environment. Currently much of the effort in genetic epidemiology is largely focused on attempting to identify which genes influence which phenotypes, largely through genomewide efforts employing either case/control study designs utilizing association methods. While the identification of the key genes involved in the expression of a phenotype, particularly for those involved in mediating disease risk, is an important endeavour, it only represents a first step. It is highly doubtful that any gene will exert its effects completely in isolation but rather will have its action modulated by a wide range of other genetic, epigenetic, and also environmental factors. The identification of such environmental factors and the deciphering of how they impact the action of genes are a fundamental objective of genetic epidemiological analyses. Therefore, as the diversity, as well as shear amount, of genetic information continues to accumulate, the thoughtful definition and quantification of key environmental factors must also keep pace if we are to truly understand how the critical interaction between genes and environment gives rise to the phenotypic variation we observe at the population level [49].

## 5. What Could Be Done?

The idea is to create intermodal transport chain between Bari Logistic Center and Logistic Railways Terminals in Balkan Region avoiding the road traffic and reduction of CO_2_ using short sea shipping by Ro/Ro vessels and block trains. One of the European Commission measures is to shift the balance between transport modes with focusing and promoting intermodal transport, type of transport strongly advocated due to its environmental concerns, safety reasons, and road congestions avoidance.

The first step is organization of railways practice in Bosnia and Herzegovina, Serbia, Romania, Montenegro, Croatia, and Bulgaria, mixing private and public consortia, which are going to be able to move merchandise from/to Southern Europe to/from Eastern Europe. It is necessary to create an Intergovernmental Working Group on Railways—new railway management model able to take care of the opportunities given by all existing European Programs on intermodal transport sector—which will include all countries interested in a project start up. The aim of EU policy is to reduce and also to eliminate technical and operational differences among national railway systems, with subsequently achieving harmonization in terms of technical specifications for infrastructure, signaling, telecommunications, and rolling stock taking care of certain operational rules [50, 51] creating common intermodal policy.

Furthermore, this idea could have operational implications in existence of more friendly transport solution, sustainable development, climate change prevention, government regulation, public education, and public policy issues related to human health by creation of health-supportive environment.

This is an integrated approach based on environmental and biological monitoring, including the analysis of biomarkers of exposure, early biological effects, and susceptibility that could be useful to evaluate global benefits (such as economics, logistics, transport, environmental, and/or climate impact) and would be the translation of emerging scientific knowledge in environmental health science into practical and useful information for clinical medicine as well as for public health policy.

## 6. Final Remarks

The influence of genetic polymorphisms of the genes encoding for detoxification enzymes on a series of biomarkers was demonstrated before [37].

Human variability, especially as it relates to polymorphisms in biotransformation enzymes, represents an important factor to consider in evaluating the effects of exposure to genotoxic substances, because polymorphisms are able to act on the individual susceptibility even to the neoplastic transformation [52].

Advances in molecular analytsis give the possibilities for understanding the genetic contribution to phenotypic outcomes, same as to develop new and creative research designs and techniques to integrate the vast amount of biological information into models and careful measurement of the environment. This will, necessarily, have to be a multidisciplinary team science approach.

The contamination of air same as climate change is essentially a social problem and because of that it needs integral and coherent transport policy. The social implications of the transport need to be constantly and carefully monitored.

The starting point is to find sustainable transport and welcome the development of infrastructure changing as a policy instrument to contain and reduce congestion, and reduce environmental impacts. It is well reported by Kreutzberger et al. [53] that the environmental performance of intermodal transport is substantially better than that of unimodal road transport when looking at every use and GHG emission and this is even more outspoken when also local emissions, accidents, congestion, and noise are integrated. As regards of the automatic link between economic growth and growth in freight transport, the solution is not in reduction of transport but in redistribution between modes. This is a main reason and strength of a project idea which could bring a success. Furthermore, in this case we are not only talking about redistribution between modes [54] of transport but also we are implementing a new corridor. Enlargement of the European Union is set to trigger larger exchanges of goods and so need for additional investments in transport infrastructures. It is well known that south-east Europe transport system distinguishes itself by extremely fragmented transport. Italy, especially South Italy, with its geographical position, cultural, political, humanitarian, and historical connections could have prestige and favorable role between European Union and Balkans. Implementation of legal regulations under supervision could produce different positive consequences on health, transport, environment, climate, and so forth.

Environmental pollution is reaching worrying proportion worldwide and solution probably could be in different small changes of lifestyle habits which, all together, will be able to produce reduction of potentially toxicants, money waste, and improvement of environmental with improvement of health and life quality.

We can find a good example in “multifactorial” diseases. “Multifactorial” recognizes that these disorders are the result of both environmental and genetic factors and does prejudge the relative role of either category. Namely, in this type of diseases when conditions are favorable, that is, different mutations and/or polymorphisms of susceptibility together with adverse environment conditions, a pathological situation is observed. However if involved genes are going to be considered singularly, no one will be able to produce pathology, but with contribution given by every gene, the interaction between them, and environment we have to deal with illnesses.

**Figure 3:**
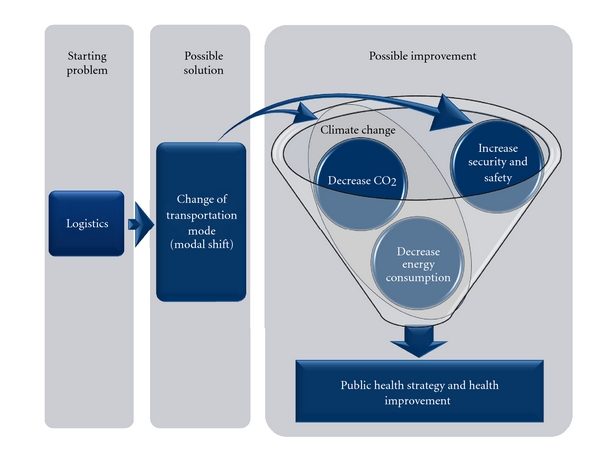
Schematic presentation of project idea. Starting from logistic problem, passing through possible solution-introduction of intermodal transport, problem solution could be achieved with improvement on climate change, security, and safety with positive effects on public health.

Finally we proposed in Figure 3 wide transdisciplinary actions of project idea, which brings a solution for economic activity such as transportation, strongly related to pollution output with possible repercussions on climate change and public health.

So, if we consider our planet as “living organism” and our action as expression of genes, then individually small actions in different fields could be extremely potent and benefits could be obtained to us and future generations.
